# Female Affective Perception of Mainstream and Paraphilic Pornography: Associations with Sexual and Psychological Intrapersonal Variables

**DOI:** 10.1007/s10508-023-02701-8

**Published:** 2023-10-20

**Authors:** Sabine Prantner, Cristina Giménez-García, Alejandro Espino-Payá, Miguel A. Escrig, Nieves Fuentes-Sánchez, Rafael Ballester-Arnal, M. Carmen Pastor

**Affiliations:** 1 Departamento de Psicología Básica, Clínica y Psicobiología, Facultad de Ciencias de la Salud, Universitat Jaume I, 12071 Castelló de la Plana, Spain; 2 Institute for Biomagnetism and Biosignalanalysis, University of Münster, Münster, Germany; 3 Departamento de Psicología. Facultad de Ciencias de la Salud, Universidad Europea de Valencia, Valencia, Spain

**Keywords:** Pornography, Valence, Arousal, Disgust, Moral and ethical acceptance, Women

## Abstract

Understanding affective perceptual processes can further contribute to the explanation of motivation and actions, as well as sexual risk behaviors. Pornography can be considered salient emotional content and is popular, also among females. Yet, the female perspective on pornography has often been overlooked and it remains unclear how individual variables may be associated with the affective perception of pornography and could provide a risk profile. Possible associations between several sexual and psychological intrapersonal variables and the affective perception of various forms of pornography were analyzed from the female perspective. A sample of 231 females (*M* = 21.87 years; SD = 3.9 years) provided ratings of affective valence, arousal, disgust, and moral and ethical acceptance for mainstream pornographic and paraphilic images of dominance, submission, or sexual violence. Paraphilic pornography was perceived as less pleasant, arousing, and moral and ethically acceptable, but more disgusting compared to mainstream pornography. This was more pronounced among females who had never consumed pornography. Results further suggest that the female affective perception of pornography was associated with the following sexual intrapersonal variables: sexual sensation seeking for physical sensations, erotophilia, lack of sexual control, problematic pornography consumption, and sexual disgust sensitivity. Of the assessed psychological intrapersonal variables, only anxiety was negatively associated with disgust for paraphilic pornography. It is important to further analyze the female affective perception of pornography and associated variables to include them in strategies for prevention and for addressing problematic consequences of the acceptance of specific sexual content and behaviors, especially related to sexualized violence.

## Introduction

Affective perception refers to the process of experiencing emotional content of any kind and can be measured on a behavioral level. Emotions may be considered powerful, pervasive, predictable, sometimes harmful and sometimes beneficial drivers of decision making and are associated with affective perceptions, routinely affecting how and what we see, and have strong motivational forces (Bradley et al., 2001; Döring, 2007; Lang et al., 1998; Lerner et al., 2015; Mirabella, 2018; Zadra & Clore, 2011). Sexual stimuli are known to be of particular emotional salience (Bradley et al., 2001). Thus, understanding affective perceptual processes can further contribute to the explanation of motivation and actions, as well as potentially risky sexual behaviors. Although a vast array of environments are available, individuals will often be selective, choosing only those environments that are best-suited to them and are congruent with their affect and/or individual dispositions (Emmons et al., 1985, 1986). This association has also been noted for sexual contexts, as people are said to be selective in their sexual media viewing habits, directing their choices to material that is congruent with preexisting dispositions (Bogaert, 2001). Along these lines, previous research has linked pornography use to several intrapersonal variables such as greater sexual motivation, sexual compulsivity, affect, and anxiety (e.g., Bőthe et al., 2019; Castro-Calvo et al., 2018; Kowalewska et al., 2022; Paul, 2009; Willoughby et al., 2019), but has not examined its relationship to females' affective perception of diverse pornographic content. Although consuming pornography has become more common for large segments of the population, also among females with a female pornography consumption between 40 and 60%, the literature focuses mainly on the male perspective (Ballester-Arnal et al., 2017, 2022; Blais-Lecours et al., 2016; Bőthe et al., 2018; Bridges et al., 2016; Grubbs et al., 2021; Martyniuk et al., 2016; Rissel et al., 2017; Zheng & Zheng, 2014).

The current study fills an important gap in the literature by examining associations between diverse sexual and psychological intrapersonal variables, and the affective perception of mainstream and paraphilic pornography among Hispanic females. This will contribute to a better comprehension of the female experience with pornography and possible intraindividual associations with it, which is particularly important for understanding preferences for and acceptance of different sexual scenarios, as well as the motivational processes involved.

### Pornography and Paraphilic Interests

The security and anonymity offered by the Internet may support the discovery and development of new preferences and sexual behaviors (Griffiths, 2012; Hald & Štulhofer, 2016; Walters & Spengler, 2016; Yu, 2013). In this regard, about 10% to 15% of pornography users are said to prefer non-mainstream pornographic content such as violent, coercive, fetish, bondage and discipline, or sadomasochistic pornography, which often is nested under the concept of paraphilic interests (Hald, 2006; Sørensen & Kjørholt, 2007; Štulhofer et al., 2007). An increase in aggressive content in pornographic videos, especially the more subtle violence, has been reported (Bridges et al., 2010; Carrotte et al., 2020; Fritz et al., 2020). Sexual fantasies of submission and domination seem to be quite common, and across different studies between 10 and 30% of people reported paraphilic interests (Bártová et al., 2021; Brown et al., 2020; Chan, 2021; Hald, 2006; Joyal et al., 2015; Sørensen & Kjørholt, 2007; Stefanska et al., 2022).

Regarding gender differences toward paraphilic preferences, little research has been conducted in this line, but males seem to show more general paraphilic interests and thus may be more likely to engage in paraphilic pornography (Chan, 2021; Stefanska et al., 2022). Nevertheless, prior research also shows that exposure to violent pornography is associated with dating violence and that the odds of exposure to such forms of violence are higher for females than for males (Herbitter et al., 2022), which highlights the importance of examining acceptance and enjoyment of violent sexual content from a female perspective.

One hypothesis is that viewing of such pornographic material can favor the acquisition of harmful sexual scripts also in offline sexual behaviors (Bridges et al., 2016; Vandenbosch & van Oosten, 2017; Wright, 2011). Violent pornography has been repeatedly linked to problematic outcomes such as sexually aggressive attitudes and behaviors (Allen et al., 1995; Bogaert, 2001; Hald et al., 2010; McKee, 2015; Seto et al., 2001). Viewing pornography may promote sexual objectification and sexually objectifying attitudes (Willis et al., 2022), and it is likely that endorsement of sexual objectification can lead to acceptance of violence against women (Wright & Tokunaga, 2016). It does not take extreme forms of sexual objectification such as sexual assault to already negatively impact those targeted (Szymanski & Feltman, 2014), whereby in the case of violent pornography most often females are targeted (Bridges et al., 2010; Carrotte et al., 2020). Already subtle, everyday sexual objectification can impair the emotional well-being of those targeted (Szymanski & Feltman, 2014).

However, only few studies have examined individual variability in sexual and affective responses to sexually violent stimuli. Previous research of this type has been conducted primarily with male samples and has shown, for example, that males with high self-reported arousal toward sexually violent movies, and individual predispositions of among others higher hypersensitivity, and high interest in sexual variety as well as a promiscuous approach to sexual activity are more prone to watch sexually violent content and to accept sexual violence (Bogaert, 2001; Fisher et al., 2013). Lower sexual inhibition proneness in males was also associated with higher subjective sexual arousal toward visual stimuli of non-consensual sexual intercourse in experimental settings (Carvalho & Rosa, 2020; Carvalho et al., 2023).

### Pornography and Associations with Intrapersonal Variables

Research suggests that a number of individual variables may play a role regarding the viewing and perception of pornographic material. Intrapersonal variables such as greater sexual motivation, sexual openness, and pleasure seeking were associated not only with paraphilic interests but also with mainstream pornography (Baranowski et al., 2019; Bogaert, 2001; Carvalho & Rosa, 2020; Carvalho et al., 2023; Castro-Calvo et al., 2018; Emmers-Sommer et al., 2013; Fisher et al., 2013; Rissel et al., 2017). Sensation seeking is commonly associated with different aspects of online sexual activities and especially pornography consumption (Bőthe et al., 2019; Zheng & Zheng, 2014).

Furthermore, an individual's sexual disposition of erotophobia versus erotophilia was found to relate to their arousal toward pornography, indicating that those with more erotophilic tendencies are more likely to approach and show greater sexual arousal in response to explicit sexual stimuli (Paul, 2009).

Also, aspects of compulsive sexual behavior and hypersexuality have been associated with pornography consumption and riskier usage patterns in particular (Clayton et al., 2018; Derbyshire & Grant, 2015; Kowalewska et al., 2022). In this sense, some users seem to develop a problematic pornography consumption pattern with strong negative intra- and interpersonal consequences, associated with, e.g., affect and emotion in terms of increased anxiety and depression (Ballester-Arnal et al., 2017, 2021, 2022; Bancroft, 2008; Castro-Calvo et al., 2018, 2021; Clayton et al., 2018; Middleton et al., 2008). Greater emotional avoidance has also been associated with problematic female pornography consumption (Baranowski et al., 2019).

Another important intrapersonal variable related to sexual behavior is disgust sensitivity. Disgust sensitivity has emerged as an important trait in personality, social, and moral psychology, primarily because disgust serves a very important function, namely regulating certain threats, such as those of infectious diseases, costly sexual behavior, and risky interactions within groups (de Jong & Merckelbach, 1998; Inbar et al., 2009; Mancini et al., 2001; Olatunji et al., 2007; Tybur et al., 2010, 2011). Disgust sensitivity has been shown to be relevant to general and mental health, but also specifically to sexual health. However, it has also been suggested that sexual arousal may in turn inhibit sexual disgust to promote, e.g., the willingness of females to engage in potentially risky behaviors (Lee et al., 2014). Thus, on the one hand, disgust sensitivity can motivate people to avoid potentially dangerous activities (Curtis et al., 2011; Oaten et al., 2009) but, on the other hand, excessive disgust might also contribute to sexual problems (de Jong et al., 2009, 2010).

Viewing sexual material has been associated with higher levels of depressive symptoms (Willoughby et al., 2019). But, the affective state can also impact an individual's ability to experience pleasurable and anxiety-free sexuality (Laurent & Simons, 2009). Research on female sexual arousal indicates that, i.e., both physiological and subjective measures of sexual arousal vary as a function of anxiety level (Kalmbach et al., 2014). A relationship between affect and anxiety with female sexual dysfunction has been discussed (Fabre & Smith, 2012; Goldstein, 2000); therefore, it may be assumed that affect and anxiety are also related to the female affective perception of pornographic material.

### Current Study

According to the aforementioned reasons, the aim of this study was to do an exploratory analysis of sexual and psychological intrapersonal variables (i.e., sexual sensation seeking, hypersexuality, problematic pornography consumption, sexual disgust sensitivity, general affect, anxiety, depression) that might be associated with the affective perception of mainstream and paraphilic pornography in a sample of Hispanic females. In relation to paraphilic pornography, this study focuses on certain forms of paraphilia, which can be counted among subgroups of violent pornography, as they primarily depict acts of sexual dominance/submission. In this context, the following research questions were addressed: (RQ1) Will females show differences in regard to their affective perception toward mainstream and paraphilic pornographic content? (RQ2) How are the aforementioned sexual and psychological intrapersonal variables associated with the female affective perception of mainstream and paraphilic pornography?

## Method

### Participants

The data were obtained within a larger research project, and data from male participants have been excluded in order to focus exclusively on the female affective perception of pornography. Inclusion criteria were female assigned at birth, over 18 years old and Spanish-speaking. Thus, a total of 231 female participants aged between 18 and 51 years (*M* = 21.87; SD = 3.9) were included in this study. The majority of the participants were college students or graduates from various faculties of universities in Spain with 15.2% vocational training, 81.8% Bachelor’s degree and 3% Master’s or Ph.D. degree. Participants self-reported their sexual orientation and identified as 68.4% heterosexual, 22.9% bisexual, 2.6% homosexual, 5.2% pansexual and 0.9% asexual. Following Kinsey et al. (1948), participants were also asked about their sexual attraction: “Please indicate which of the following statements best describes to whom you are sexually attracted.” The participants could answer this question on a scale from 1 = *I am attracted only to the other sex* to 7 = *I am attracted only to my own sex*. Additionally, the following option was offered: 0 = *I am not attracted to any sex*, whereby no female of the analytic sample chose this option. Participants were asked to indicate choosing between *Yes* and *No* if they have had same-sex experiences, had ever consumed pornography voluntarily or were currently in a romantic relationship. The majority has not had same-sex experiences (84.42%), but had consumed pornography voluntarily (74.46%). Further, 54.98% reported being in a romantic relationship. Additionally, participants were asked about their religious beliefs and how conservative they considered their sexual education on a scale from 1 = *very conservative* to 7 = *very open*. See Table 1 for a reporting of descriptive statistics for the study sample.

**Table 1:** Descriptive statistics of participants’ characteristics

	*n*	% or mean (*SD*)
*Level of education*		
Vocational training	35	15.2
Bachelor’s degree	189	81.8
Master’s or PhD degree	7	3.0
*Gender identity*		
Woman	226	97.8
Agender	5	2.2
*Self-reported sexual orientation*		
Heterosexual	158	68.4
Bisexual	53	22.9
Homosexual	6	2.6
Pansexual	12	5.2
Asexual	2	0.9
Sexual attraction^a^	231	2.24 (1.48)
*Same-sex experiences*		
Yes	36	15.58
No	195	84.42
*Ever consumed pornography*		
Yes	172	74.46
No	59	25.54
*Romantic relationship*		
Yes	127	54.98
No	104	45.02
*Religious beliefs*		
Practicing believer	23	10.04
Non-practicing believer	57	24.89
Atheist or agnostic	149	65.07
^b^Conservatism of sexual education	231	3.11 (1.22)

^a^Sexual attraction by Kinsey et al. (1948): 1 = *I am attracted only to the other sex* to 7 = *I am attracted only to my own sex,* ^b^Conservatism of Sexual education: 1 = *very conservative* to 7 = *very open*

### Procedure

The present study is part of a larger investigation of sexual perception that aims to standardize an explicit pornographic picture set. Due to the sanitary health crisis inflicted by COVID-19, the assessment procedure took place via online meetings using Google Meet and Qualtrics. Sessions lasted approximately 90 min. Several recruitment channels were used inviting individuals over 18 years old to an online study about the perception of diverse explicit pornographic pictures, including campus advertisement, social media and personal communication in the form of invitations to friends, colleagues and acquaintances of the researchers. Individuals interested in the study made appointments with the principal investigator to participate. During the assessment procedure, participants were first informed of the anonymous, voluntary and confidential nature of this study and then given the instructions to how the affective ratings to the pornographic stimuli are correctly done by two experimenters followed by several practice trials in order to make sure that the procedure was understood. Following the affective ratings, participants filled in the sociodemographic and sexuality-related questions, as well as the questionnaires assessing the intrapersonal variables of interest. The participants took part using their private devices (computers, tablets or laptops). During the whole procedure, participants were able to ask the two experimenters via the chat of Google Meet for private assistance when in doubt.

In total, 192 pornographic pictures acquired from online pornography platforms and 24 control IAPS pictures were rated by the participants (Lang et al., 1999). The pornographic images belong to an explicit pornographic picture set, which is currently being standardized on a Spanish-speaking population and available upon request. These pictures were prior gathered by females from feminist (ErikaLust) and amateur (flickr) pornography platforms, when needed permission to use for research purposes has been acquired by the producers, and eventually categorized by six experts (50% females) in the field of sexuality according to the sexual practices and the sexes of the pornography performers depicted.

Eventually, 72 images—from four sets of each different 48 sexually explicit pornographic images and the same 24 IAPS control stimuli—were presented to each participant. Each pornographic picture was rated by an average of 57 females. The stimuli consisted of equal-sized images of the different categories of opposite- and same-sex interactions of most common and also popular sexual practices in mainstream pornography: masturbation, oral sex, vaginal sex, anal sex, group sex, and additionally images outside mainstream pornography, which are of paraphilic media showing scenes of dominance, submission, sexual violence or fetishes (8 images per category). This categorization was based on Hald and Štulhofer (2016). For this study, a total of 5 images from the category of paraphiliac pornography was excluded from further analysis, because these images showed fetish content (e.g., transvestism, foot fetish) and did not picture dominance, submission or sexual violent pornography. Stimuli were presented in randomized order for each participant using the Qualtrics randomization tool.

### Measures

#### Affective Ratings

The images were presented one at a time during 6 s each, followed by the subjective ratings. The emotional dimensions of affective valence and arousal were assessed using a 9-point non-verbal Self-Assessment Manikin rating scales (SAM; Bradley & Lang, 1994), where 1 = *highly unpleasant*/ *least arousing*, and 9 = *highly pleasant*/*highly arousing,* respectively. In addition, the emotional categories of disgust as well as moral and ethical acceptance were dimensionally rated using 9-point Likert rating scales. The disgust scale ranged from 1 = *not at all disgusting* to 9 = *extremely disgusting*. The moral and ethical acceptance scale ranged from 1 = *absolutely immoral* to 9 = *totally acceptable*. Participants were asked to give an immediate, spontaneous reaction to the seen images. Each rating scale was available for 6 s before skipping to the next one. After 2 s, participants also had the option to click on a next-button in order to see the next rating scale. As soon as the participants finished all the ratings of one image, a new screen with the subsequent randomized image was displayed preceded by an alert message. The affective ratings were presented in a randomized order to avoid halo effects.

#### Sexual Intrapersonal Variables

##### Sexual Sensation Seeking

The Sexual Sensation Seeking Scale (SSSS) measures sensation seeking related to sexual behavior (Kalichman et al., 1994). The SSSS includes 11 items with 4-point scales (1 = *not characteristic of me* to 4 = *very characteristic of me*) and can be analyzed in two subscales of physical sensations attraction (PSA), referring to the emphasis on the physical and sensual aspects of sexual encounters and the desire to feel sexual stimulation, and new experiences seeking (NES), related to the desire to try new sexual experiences (Kalichman et al., 1994). That structure was later also verified for a Spanish sample, with high internal consistency for the total scale (*α* = 0.81), as for each factor (Ballester-Arnal et al., 2018). Moreover, the SSSS had adequate convergent validity, making it a useful brief screening measure in research for Spanish-speaking people (Ballester-Arnal et al., 2018). In the current study, Cronbach’s alpha was 0.75 for the total scale, 0.64 for PSA and 0.84 for NES.

##### Erotophilia

The EROS is an adaptation of the Sexual Opinion Survey and measures the bipolar concept of erotophilia–erotophobia, which refers to learned attitudes toward sexual scenarios along a continuum that extends from a negative pole (erotophobia) to a positive pole (erotophilia) (del Río Olvera et al., 2013). Higher scores indicate more erotophilia and lower scores more erotophobia. Thus, individuals who score high on erotophobia would tend to respond with more negative attitudes to sexual scenarios, evaluating them negatively and trying to avoid them. On the other hand, people who score high on erotophilia would have the opposite behavior, i.e., they would respond with more positive attitudes to sexual scenarios, evaluate them positively and seek those out. It consists of 20 items which are answered on a 7-point scale (1 = *totally disagree* to 7 = *totally agree*). The EROS has adequate parameters, like a high reliability with an internal consistency of 0.85, for use in research in Spanish samples (del Río Olvera et al., 2013). In the present study Cronbach's alpha was also 0.85.

##### Hypersexuality

The Hypersexual Behavior Inventory (HBI) is a questionnaire composed of three subscales, assessing coping (sex and sexual behaviors as a response to emotional distress), control (lack of self-control in sexuality-related behaviors), and consequences (diverse consequences of sexual thoughts, urges, and behaviors) associated with sexual thoughts, feelings and behaviors related to hypersexuality (Reid et al., 2011). It consists of 19 items with 5-point scales (1 = *never* to 5 = *many times*). The psychometric properties of the HBI suggest it has high reliability and validity ranging between 0.89 and 0.96 also for Spanish speakers (Ballester-Arnal et al., 2019). In the present study, Cronbach’s alpha was 0.88 for the total scale, and 0.86 for coping, 0.76 for control, and 0.69 for consequences.

##### Problematic Pornography Consumption

The PPCS-6 (Short Version of the Problematic Pornography Consumption Scale) assesses problematic pornography use measuring salience, tolerance, mood, modification, relapse, withdrawal, and conflict (Bőthe et al., 2021). It includes 6 items that are answered on a 7-point scale (1 = *never* to 7 = *all the time*). The PPCS-6 yielded strong psychometric properties and offers a cutoff score of 20 out of 42 points to distinguish between problematic and non-problematic pornography use. With Cronbach’s alpha indices higher than 0.70, the PPCS-6 can be considered a reliable and valid scale to assess PPU in studies (Bőthe et al., 2021). In the present study, Cronbach’s alpha was 0.79.

##### Sexual Disgust Sensitivity

The Spanish questionnaire Escala Multidimensional de Sensibilidad al Asco/Multidimensional Disgust Scale (EMA) is a 30-item multidimensional scale of disgust, whereby one subscale of 5 items measures sexual disgust on a 5-point scale (1 = *not at all* to 5 = *very much*) (Sandín et al., 2013). It showed good reliability and the internal consistency of this subscale is high (Sandín et al., 2013). Cronbach’s alpha in this study was 0.71.

#### Psychological Intrapersonal Variables

##### Positive and Negative Affect

The Positive and Negative Affect Schedule (PANAS) scales assess positive and negative affect via each 10 items with 5-point scales (1 = *very slightly or not at all* to 4 = *extremely*) (Watson et al., 1988). Support is provided for the two-dimensional structure of the measure (i.e., positive and negative affect), as well as for its high internal consistency, reliability, and stability over time, also within Spanish-speaking samples with Cronbach’s alpha higher than 0.70 (Sandín, 2003; Watson et al., 1988). Regarding the internal consistency in the present study, Cronbach’s alpha is 0.91 for positive affect and 0.89 for negative affect.

##### Anxiety and Depression

The Hospital Anxiety and Depression Scale (HADS) has been developed to be a reliable instrument for detecting states of depression and anxiety in the setting of outpatient samples (Zigmond & Snaith, 1983). It comprises 14 items with 4-point rating scales, ranging from 0 to 3. Also, the Spanish version of the HADS had good internal consistency and external validity, with favorable sensitivity and specificity in identifying anxiety and depression (Herrero et al., 2003). The internal consistency, as assessed by Cronbach’s alpha, was 0.90 for the full scale, 0.84 for the Depression subscale and 0.85 for the Anxiety subscale (Herrero et al., 2003). For the present study, Cronbach’s alpha was 0.90 for the full scale, 0.77 for the Depression subscale and 0.86 for the Anxiety subscale.

### Data Analysis

Data were analyzed with R using RStudio Desktop (RStudio Inc., Boston, MA, USA). Descriptive statistics summarized study variables.

Bivariate correlation matrix of the intrapersonal variables of interest (i.e., sexual sensation seeking, erotophilia, hypersexuality, problematic pornography consumption, sexual disgust sensitivity, affect, anxiety, depression) were calculated. Affective ratings (i.e., valence, arousal, disgust, moral and ethical acceptance) given for the mainstream and paraphilic pornographic images were further compared using paired *t* tests, and effect sizes of Cohen’s *d* were calculated. Because partnership status and previous experiences with pornography were expected to influence one's affective ratings to explicit content (Kunaharan et al., 2017), Welch’s *t* tests and effect sizes of Cohen’s *d* were calculated regarding these binary variables for all affective ratings (i.e., valence, arousal, disgust, moral and ethical acceptance) assessed. Affective ratings did not differ significantly between groups of participants, who had and those who had not reported a romantic partnership (*p* > 0.05). However, affective ratings differed significantly between groups of participants, who have and those who have never consumed pornography before (*p* < 0.05). Consequently, for the further analyses previous pornography consumption has been introduced as covariate. Additionally, self-reported sexual attraction has been included as a covariate in all multiple linear regression models, controlling like this for the different scenarios of same- and opposite-sex content in the pornography stimuli. A series of multiple linear regression models were conducted with the aim of exploring the effect of variables of interest (i.e., sexual sensation seeking, erotophilia, hypersexuality, problematic pornography consumption, sexual disgust sensitivity, affect, anxiety, depression) with regard to the different affective ratings to the mainstream pornographic images. Then, this process was repeated for the affective ratings given to the paraphilic images.

All variables of interest were *z*-standardized before the final analyses and assessed for multicollinearity. Diagnostic plots of residuals were used to assess the validity of multiple regression assumptions. The significance threshold was set to *α* = 0.05. All analyses should be regarded as exploratory.

## Results

### Descriptive Analysis of Intrapersonal Variables

In Table 2, the participants’ questionnaire data regarding their intrapersonal variables of interest are described. Within this sample, less than 2% of respondents had reported actual problematic pornography use according to PPCS-6 cutoffs (Bőthe et al., 2021).

**Table 2:** Questionnaire descriptive statistics

	*n*	*M*	95% CI	SD	Min	Max
*Sexual sensation seeking (SSSS)*						
Physical sensations attraction	231	16.01	15.51–16.51	3.83	8	27
New experiences seeking	231	9.33	9.01–9.65	2.47	3	12
Erotophilia (EROS)	225	108.9	106.62–111.19	17.49	20	140
*Hypersexual behavior (HBI)*						
Coping	231	12.11	11.47–12.76	4.97	7	34
Control	230	10.44	10.03–10.93	3.45	8	26
Consequences	230	5.03	4.77–5.26	1.91	4	20
Problematic pornography consumption (PPCS-6)	231	8.62	8.15–9.10	3.69	6	23
Sexual disgust sensitivity (EMA)	231	10.42	9.94–10.90	3.67	5	25
*Positive and negative affect (PANAS)*						
Positive affect	231	32.58	31.50–33.65	8.26	10	50
Negative affect	231	23.21	22.17–24.25	8.03	10	50
*Anxiety and depression (HADS)*						
Depression	231	8.3	7.73–8.87	4.4	0	20
Anxiety	231	5.72	5.22–6.22	3.9	0	17

SSSS, Sexual Sensation Seeking Scale (ranging from 1 to 4); *EROS*, adaptation of the Sexual Opinion Survey (ranging from 1 to 7); HBI, Hypersexual Behavior Inventory (ranging from 1 to 5), PPCS-6, short version of the Problematic Pornography Consumption Scale (ranging from 1 to 7); EMA, Escala Multidimensional de Sensibilidad al Asco/Multidimensional Disgust Scale (ranging from 1 to 5); PANAS, Multidimensional Disgust Scale (ranging from 1 to 4); HADS, Multidimensional Disgust Scale (ranging from 0 to 3)

Table 3 includes the correlation matrix between the intrapersonal variables of interest.

**Table 3:** Correlation matrix of intrapersonal variables

	1	2	3	4	5	6	7	8	9	10	11	12
1. Physical sensations attraction (SSSS)	1.00											
2. New experiences seeking (SSSS)	0.42***	1.00										
3. Erotophilia (EROS)	0.57***	0.56***	1.00									
4. Coping (HBI)	0.40***	0.32***	0.32***	1.00								
5. Control (HBI)	0.30***	0.12	0.19**	0.48***	1.00							
6. Consequences (HBI)	0.24***	0.13*	0.14*	0.45***	0.48***	1.00						
7. Problematic pornography consumption (PPCS-6)	0.49***	0.16*	0.32***	0.37***	0.32***	0.25***	1.00					
8. Sexual disgust sensitivity (EMA)	− 0.27***	− 0.24***	− 0.52***	− 0.12	− 0.11	− 0.11	− 0.12	1.00				
9. Positive affect (PANAS)	0.17***	0.07	0.01	− 0.05	− 0.13*	0.04	0.05	0.00	1.00			
10. Negative affect (PANAS)	0.12	0.14*	0.09	0.36***	0.36***	0.21***	0.21***	0.08	− 0.27***	1.00		
11. Anxiety (HADS)	0.06	0.16*	0.10	0.33***	0.25***	0.16*	0.14*	0.03	− 0.41***	0.77***	1.00	
12. Depression (HADS)	0.03	0.15*	0.01	0.35***	0.31***	0.19***	0.10	0.03	− 0.51***	0.61***	0.76***	1.00

SSSS, Sexual Sensation Seeking Scale; EROS, adaptation of the Sexual Opinion Survey; HBI, Hypersexual Behavior Inventory; PPCS-6, short version of the Problematic Pornography Consumption Scale; EMA, Escala Multidimensional de Sensibilidad al Asco/Multidimensional Disgust Scale; PANAS, Positive and Negative Affect Schedule; Multidimensional Disgust Scale; HADS, Multidimensional Disgust Scale. *p < .05; **p < .01; ***p < .001

### Differential Analysis of Mainstream and Paraphilic Pornography

Table 4 depicts mean and standard deviation values of the affective ratings to the different pornography content as well as inferential statistics and effect sizes. The affective ratings of the participants are indicating that the mainstream and paraphilic category were separated in the arousal, valence, disgust and moral and ethical acceptance spaces.

**Table 4:** Differential analysis of affective ratings to mainstream and paraphilic pornography

	Mainstream				Paraphilic				Inferential statistic			Effect size
	*M*	SD	Min	Max	*M*	SD	Min	Max	*t*	df	*p*	*d*
Arousal	5.37	1.58	1	8.8	4.93	1.60	1	9	6.50	227	< .001***	0.43
Valence	5.87	1.21	1.9	8.4	4.33	1.40	1	8.4	18.76	227	< .001***	1.24
Disgust	2.23	1.33	1	7.3	3.14	1.73	1	9	− 10.67	230	< .001***	0.70
Moral and ethical acceptance	7.85	1.39	2.7	9	6.38	1.71	2	9	18.04	230	< .001***	1.12

*p < .05; **p < .01; ***p < .001

Mean arousal ratings of paraphilic pictures were significantly lower compared to mainstream pornography for the total sample (*t*(227) = 6.5, *p* < .001, *d* = 0.43). Females were found to show significantly more valence for mainstream than paraphilic pornography (*t*(227) = 18.76, *p* < .001, *d* = 1.24). They found mainstream less disgusting (*t*(230) = − 10.67, *p* < .001, *d* = 0.70) and more morally and ethically acceptable (*t*(230) = 18.04, *p* < .001, *d* = 1.12) than paraphilic pornography.

**Fig. 1:**
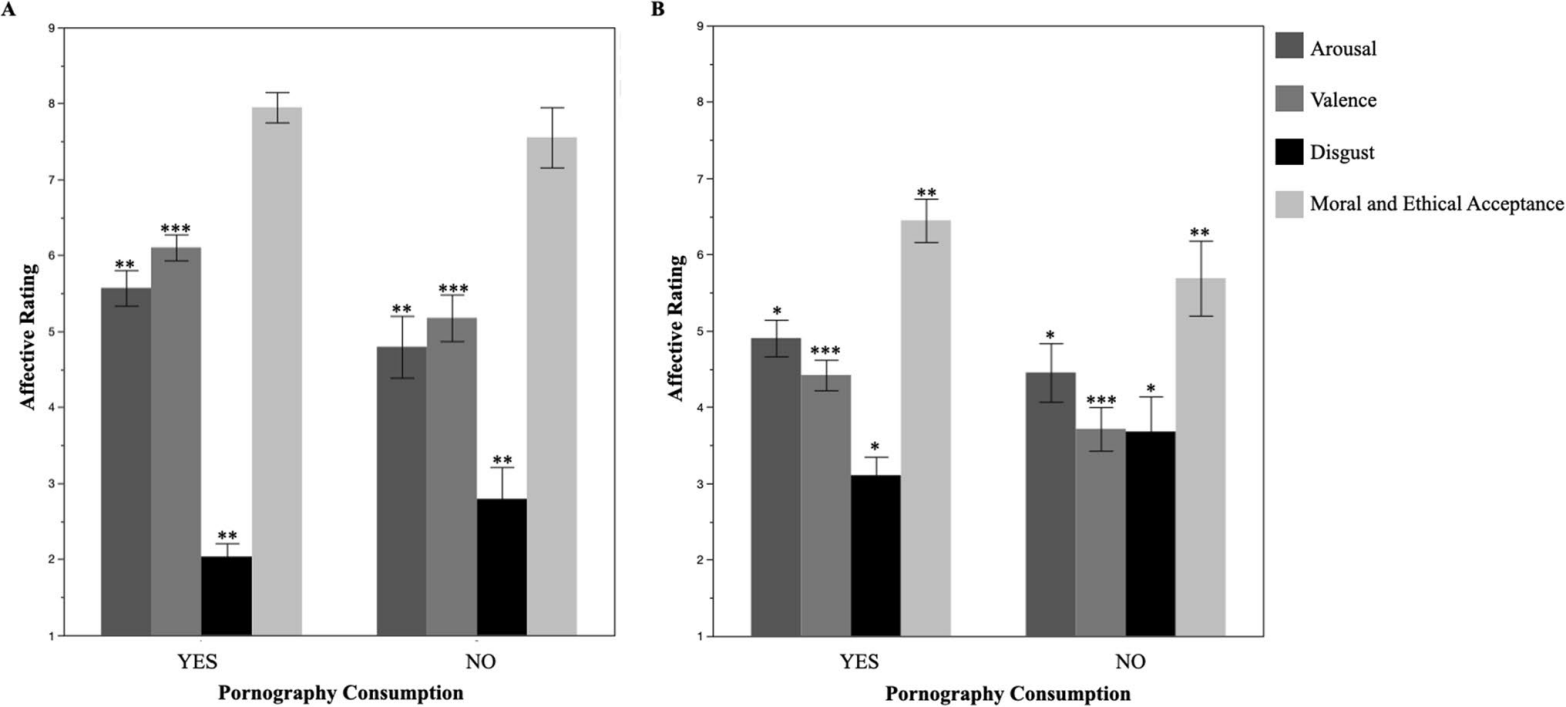
Comparison of affective ratings to **A** mainstream and **B** paraphilic pornography between females who have and those who have never consumed pornography. *Note* **A** mainstream pornography. **B** Paraphilic Pornography. Error bars were calculated as 95% CI. *p < .05; **p < .01; ***p < .001

Furthermore, females who have and who have never consumed pornography were compared regarding their affective ratings toward mainstream and paraphilia pornography (Fig. 1). Significant differences were found between these groups in mainstream pornography regarding arousal (*t*(98.51) = 3.29, *p* = .001, *d* = 0.50), valence (*t*(95.92) = 5.27, *p* < .001, *d* = 0.81) and disgust (*t*(79.8) = − 3.34, *p* = .001, *d* = 0.60). Regarding paraphilia pornography, significant differences between females who have and those who have never consumed pornography were found regarding arousal (*t*(104.31) = 2.23, *p* = .028, *d* = 0.33), valence (*t*(125.44) = 4.48, *p* < .001, *d* = 0.60), disgust (*t*(95.02) = − 2.50, *p* = .014, *d* = 0.39) and moral and ethical acceptance (*t*(98.99) = 2.87, *p* = .005, *d* = 0.44). Females, who had consumed pornography, had higher arousal, valence and moral and ethical acceptance and lower disgust ratings than females, who had never consumed pornography.

### Intrapersonal Variables and Affective Processing of Mainstream Pornography

In Table 5, four multiple regression models are presenting relevant linear model parameters for the intrapersonal variables of interest—sexual sensation seeking, erotophilia, hypersexuality, problematic pornography consumption, sexual disgust sensitivity, affect, anxiety, depression—regarding mainstream pornography affective perception. Checking for the variance inflation factors for all intrapersonal variables indicated that there was no problem with multicollinearity.

**Table 5:** Multiple linear regression analysis of intrapersonal variables on affective ratings to mainstream pornography

	Arousal			Valence			Disgust			Ethical and Moral Acceptance		
	*B* (SE)	95% CI	*p*	*B* (SE)	95% CI	*p*	*B* (SE)	95% CI	*p*	*B* (SE)	95% CI	*p*
Physical Sensations Attraction (SSSS)	**0.41 (0.13)**	**0.14 to 0.67**	**.003**	0.18 (0.09)	− 0.00 to 0.35	.053	− 0.01 (0.10)	− 0.22 to 0.19	.911	0.20 (0.12)	− 0.03 to 0.43	.096
New Experiences Seeking (SSSS)	− 0.08 (0.12)	− 0.33 to 0.17	.528	0.09 (0.08)	− 0.08 to 0.26	.278	0.01 (0.10)	− 0.18 to 0.21	.883	− 0.06 (0.11)	− 0.28 to 0.16	.596
Erotophilia (EROS)	0.20 (0.15)	− 0.10 to 0.50	.188	**0.37 (0.10)**	**0.17 to 0.57**	**<.001**	− **0.61 (0.12)**	− **0.84 to **− **0.37**	**<.001**	**0.56 (0.13)**	**0.30 to 0.82**	**<.001**
Coping (HBI)	0.05 (0.13)	− 0.20 to 0.31	.684	− 0.02 (0.09)	− 0.19 to 0.15	.811	0.12 (0.10)	− 0.07 to 0.32	.217	0.01 (0.11)	− 0.21 to 0.23	.930
Control (HBI)	− 0.05 (0.13)	− 0.30 to 0.20	.682	− 0.14 (0.09)	− 0.30 to 0.03	.108	0.19 (0.10)	− 0.00 to 0.38	.054	− **0.25 (0.11)**	− **0.47 to **− **0.03**	**.024**
Consequences (HBI)	− 0.03 (0.12)	− 0.26 to 0.20	.779	0.03 (0.08)	− 0.13 to 0.18	.723	− 0.07 (0.09)	− 0.25 to 0.10	.423	0.02 (0.10)	− 0.18 to to 0.22	.850
Problematic Pornography Consumption (PPCS-6)	0.11 (0.13)	− 0.14 to 0.36	.382	**0.20 (0.09)**	**0.03 to 0.37**	**.019**	− 0.06 (0.10)	− 0.26 to 0.13	.505	− 0.14 (0.11)	− 0.36 to to 0.07	.185
Sexual Disgust Sensitivity (EMA)	− 0.15 (0.11)	− 0.38 to 0.07	.182	− **0.23 (0.08)**	− **0.38 to **− **0.08**	**.003**	**0.28 (0.09)**	**0.11 to 0.45**	**.002**	− 0.11 (0.10)	− 0.30 to 0.09	.277
Positive Affect (PANAS)	0.03 (0.12)	− 0.22 to 0.27	.836	0.00 (0.08)	− 0.16 to 0.16	.977	0.02 (0.09)	− 0.17 to 0.20	.868	− 0.10 (0.10)	− 0.30 to 0.11	.349
Negative Affect (PANAS)	0.21 (0.16)	− 0.11 to 0.52	.197	0.06 (0.11)	− 0.15 to 0.28	.556	0.05 (0.12)	− 0.19 to 0.30	.666	0.16 (0.14)	− 0.11 to 0.44	.247
Anxiety (HADS)	− 0.06 (0.19)	− 0.43 to 0.32	.759	0.07 (0.13)	− 0.18 to 0.32	.605	− 0.04 (0.15)	− 0.33 to 0.25	.798	− 0.04 (0.17)	− 0.36 to 0.29	.829
Depression (HADS)	− 0.12 (0.17)	− 0.45 to 0.21	.480	− 0.12 (0.11)	− 0.35 to 0.10	.290	0.00 (0.13)	− 0.25 to 0.26	.983	− 0.12 (0.15)	− 0.41 to 0.17	.411
*R*^2^ / *R*^2^ adj.	0.20 / 0.14			0.42 / 0.38			0.36 / 0.31			0.25 / 0.20		
	***F*(14, 205) = 3.60, *p* < 0.001**			***F*(14, 205) = 10.51, *p* < 0.001**			***F*(14, 208) = 8.22, *p* < 0.001**			***F*(14, 208) = 4.89, *p* < 0.001**		

Controlling for covariates of previous pornography consumption and sexual attraction of the participants. SSSS, Sexual Sensation Seeking Scale; EROS, adaptation of the Sexual Opinion Survey; HBI, Hypersexual Behavior Inventory; PPCS-6, short version of the Problematic Pornography Consumption Scale; EMA, Escala Multidimensional de Sensibilidad al Asco/Multidimensional Disgust Scale; PANAS, Positive and Negative Affect Schedule; Multidimensional Disgust Scale; HADS, Multidimensional Disgust Scale. Bolded statistics indicate statistical significance (*p* < .05)

Regarding arousal, a statistically significant positive relation was found with females reporting being more aroused by this content as their PSA scores (*B* = 0.41, *p* = .003) were higher. This model explained 14% of the arousal variance for mainstream pornography (*R*^2^ = 0.20, Adj *R*^2^ = 0.14). Among the intrapersonal variables entered in the regression model for explaining the level of valence, erotophilia (*B* = 0.31, *p* < .001) and problematic pornography consumption (*B* = 0.20, *p* = .019) scores were significantly positively associated with it. Also, a negative association regarding sexual disgust sensitivity (*B* = − 0.23, *p* = .003) scores was found for the valence ratings given to mainstream pornography. This model explained 38% of the valence variance for mainstream pornography (*R*^2^ = 0.42, Adj *R*^2^ = 0.38). Erotophilia (*B* = − 0.61, *p* < .001) was negatively related to females' affective ratings of disgust to the mainstream pornographic images. Additionally, sexual disgust sensitivity (*B* = 0.28, *p* = .002) scores showed positive associations with females' affective ratings of disgust. This model explained 31% of the disgust variance for mainstream pornography (*R*^2^ = 0.36, Adj *R*^2^ = 0.31). Regarding moral and ethical acceptance ratings to pornographic images, it was found that erotophilia (*B* = 0.56, *p* < .001) was positively related to it. Additionally, sexual disgust sensitivity (*B* = − 0.25, *p* = .024) was negatively associated with moral and ethical acceptance ratings. This model explained 20% of the moral and ethical acceptance variance for mainstream pornography (*R*^2^ = 0.25, Adj *R*^2^ = 0.20).

### Intrapersonal Variables and Affective Processing of Paraphilic Pornography

The multiple regression analyses were repeated for the affective ratings toward paraphilic pornography images as outcomes. In Table 6, the regression models are presented showing relevant linear model parameters for the intrapersonal variables of interest. The variance inflation factors for all intrapersonal variables of the models were indicating that there was no problem with multicollinearity.

**Table 6:** Multiple linear regression analysis of intrapersonal variables on affective ratings to paraphilic pornography

	Arousal			Valence			Disgust			Ethical and Moral Acceptance		
	*B* (*SE*)	95% CI	*p*	*B* (*SE*)	95% CI	*p*	*B* (*SE*)	95% CI	*p*	*B* (*SE*)	95% CI	*p*
Physical Sensations Attraction (SSSS)	0.05 (0.14)	− 0.24 to 0.33	.755	− 0.19 (0.11)	− 0.43 to 0.04	.105	0.14 (0.14)	− 0.15 to 0.43	.330	− 0.03 (0.11)	− 0.38 to 0.31	.862
New Experiences Seeking (SSSS)	− 0.11 (0.13)	− 0.37 to 0.15	.405	0.15 (0.11)	− 0.07 to 0.37	.168	− 0.20 (0.13)	− 0.47 to 0.07	.151	0.23 (0.11)	− 0.09 to 0.56	.158
Erotophilia (EROS)	0.32 (0.16)	− 0.00 to 0.65	.053	0.25 (0.09)	− 0.01 to 0.52	.061	**− 0.57 (0.12)**	**− 0.91 to − 0.24**	**.001**	**0.40 (0.09)**	**0.00 to 0.79**	**.049**
Coping (HBI)	0.11 (0.14)	− 0.16 to 0.39	.419	0.13 (0.11)	− 0.09 to 0.36	.249	0.04 (0.15)	− 0.24 to 0.32	.785	− 0.05 (0.12)	− 0.38 to 0.28	.769
Control (HBI)	− 0.01 (0.13)	− 0.28 to 0.26	.961	− 0.05 (0.11)	− 0.27 to 0.17	.676	0.10 (0.14)	− 0.17 to 0.38	.470	− 0.13 (0.12)	− 0.46 to 0.19	.418
Consequences (HBI)	− 0.04 (0.12)	− 0.29 to 0.20	.728	0.06 (0.10)	− 0.14 to 0.26	.530	− 0.15 (0.13)	− 0.40 to 0.10	.248	0.10 (0.11)	− 0.20 to 0.40	.502
Problematic Pornography Consumption (PPCS-6)	0.20 (0.14)	− 0.07 to 0.47	.145	**0.32 (0.11)**	**0.10 to 0.54**	**.004**	− 0.03 (0.14)	− 0.30 to 0.24	.825	− 0.02 (0.11)	− 0.34 to 0.30	.915
Sexual Disgust Sensitivity (EMA)	− 0.06 (0.12)	− 0.31 to 0.18	.600	**− 0.26 (0.09)**	**− 0.46 to − 0.07**	**.009**	0.20 (0.12)	− 0.05 to 0.44	.118	− 0.23 (0.09)	− 0.52 to 0.07	.127
Positive Affect (PANAS)	0.03 (0.13)	− 0.23 to 0.28	.843	− 0.06 (0.11)	− 0.27 to 0.15	.595	0.05 (0.13)	− 0.20 to 0.31	.682	− 0.27 (0.11)	− 0.58 to 0.04	.083
Negative Affect (PANAS)	0.20 (0.17)	− 0.14 to 0.54	.256	0.13 (0.14)	− 0.14 to 0.41	.340	− 0.06 (0.18)	− 0.41 to 0.29	.729	0.26 (0.15)	− 0.15 to 0.67	.218
Anxiety (HADS)	− 0.01 (0.20)	− 0.41 to 0.39	.958	− 0.26 (0.17)	− 0.59 to 0.07	.162	**0.45 (0.21)**	**0.04 to 0.86**	**.033**	− 0.48 (0.17)	− 0.97 to 0.01	.055
Depression (HADS)	− 0.09(0.18)	− 0.45 to 0.27	.608	− 0.03 (0.15)	− 0.32 to 0.27	.850	− 0.13 (0.18)	− 0.50 to 0.23	.472	− 0.05 (0.15)	− 0.49 to 0.38	.802
*R*^2^ / *R*^2^ adj.	0.10 / 0.04			0.24 / 0.19			0.21 / 0.16			0.16 / 0.10		
	*F*(14, 205) = 1.77, *p* = 0.05			***F*(14, 205) = 4.64, *p* < 0.001**			***F*(14, 208) = 4.04, *p* < 0.001**			***F*(14, 208) = 2.77, *p* < 0.001**		

Controlling for covariates of previous pornography consumption and sexual attraction of the participants. SSSS, Sexual Sensation Seeking Scale; EROS, adaptation of the Sexual Opinion Survey; HBI, Hypersexual Behavior Inventory; PPCS-6, short version of the Problematic Pornography Consumption Scale; EMA, Escala Multidimensional de Sensibilidad al Asco/Multidimensional Disgust Scale; PANAS, Positive and Negative Affect Schedule; HADS, Multidimensional Disgust Scale. Bolded statistics indicate statistical significance (*p* < .05)

No significant associations between intrapersonal variables and the level of arousal toward paraphilic pornography were found in the regression model. For the affective ratings of valence ratings to paraphilic pornography, significant positive associations were found with problematic pornography consumption (*B* = 0.32, *p* = .004) scores and negative associations with sexual disgust sensitivity (*B* = − 0.46, *p* = .009). This model explained 19% of the valence variance for paraphilic pornography (*R*^2^ = 0.24, Adj *R*^2^ = 0.19). Regarding disgust perception for paraphilic pornography, negative associations with erotophilia scores were found (*B* = − 0.57, *p* = .001). Additionally, females reported more disgust for paraphilic pornography as their anxiety (*B* = 0.45, *p* = .033) was higher. This model explained 16% of the disgust variance for paraphilic pornography (*R*^2^ = 0.21, Adj *R*^2^ = 0.16). To the moral and ethical acceptance of paraphilic pornography, positive associations with erotophilia scores were found (*B* = 0.40, *p* = .049). The model explained 10% of the moral and ethical acceptance variance for paraphilic pornography (*R*^2^ = 0.16, Adj *R*^2^ = 0.10).

## Discussion

The current research aimed to explore the relation between female sexual and psychological intrapersonal variables with the affective perception of mainstream and paraphilic pornography among a Hispanic sample. This study fills several research gaps in the literature. First, only a few studies have investigated the affective perception of pornographic material comparing subjective ratings to visual sexual stimuli of mainstream pornographic and paraphilic content. Far fewer have taken a nuanced look at multiple affective dimensions in combination with a variety of meaningful intrapersonal variables that might interact with such affective perceptual processes. Moreover, this study is filling a research gap as it describes the experiences with mainstream pornographic and paraphilic content of Hispanic females, an underrepresented group in sexuality research.

Overall, the results regarding the first research question showed that mainstream and paraphilic pornography evoke different affective states. The differences suggest that females evaluate mainstream pornography more positively. They perceived this material as more pleasant, more arousing, less disgusting and reported more moral and ethical acceptance for mainstream pornographic scenarios than for paraphilic images of dominance, submission and sexual violence. This may be related to the prevalence of such interests and gender differences toward paraphilic preferences, indicating higher paraphilic interests for males (Bártová et al., 2021; Chan, 2021; Hald, 2006; Joyal et al., 2015; Sørensen & Kjørholt, 2007; Stefanska et al., 2022; Štulhofer et al., 2007). Further differences were observed regarding previous pornography consumption, females who have never previously consumed pornography showed significantly lower ratings of arousal and valence, and higher ratings of disgust, toward mainstream and paraphilic pornography. Additionally, females who have never consumed pornography rated paraphilic pornography as less morally and ethically acceptable. Pornography consumers are reporting more sexual experiences, sexual openness and higher valence ratings toward sexual stimuli than non-consumers (Emmers-Sommer et al., 2013; Kunaharan et al., 2017; Mattebo et al., 2016), which may explain the differences seen in this study between females with experiences with pornography and those without.

In relation to the second research question, the findings suggest that several intrapersonal variables are associated with the affective perception of pornography. Sexual sensation seeking of physical sexual encounters, erotophilia, lack of self-control in sexuality-related behaviors, problematic pornography consumption and sexual disgust sensitivity were associated with the affective perception of mainstream pornography. Regarding the affective perception of paraphilic pornography, significant associations were found with erotophilia, problematic pornography consumption, sexual disgust sensitivity and anxiety.

### Sexual Intrapersonal Variables

Regarding mainstream pornography, more sexual sensation seeking of physical and sensual aspects of sexual encounters and the desire to feel sexual stimulation was associated with higher arousal ratings. For paraphilic pornography, however, no associations with aspects of sexual sensation seeking were found. Further, there were no aspects of sexual sensation seeking related to how pleasant either type of pornography was perceived. General and sexual sensation seeking have previously been associated with mainstream pornography as well as with paraphilic interests (Baranowski et al., 2019; Bogaert, 2001; Bőthe et al., 2019; Castro-Calvo et al., 2018; Rissel et al., 2017). It is not surprising that a person with a higher desire to feel sexual stimulation is also feeling more aroused when exposed to images of diverse mainstream sexual practices. Such a heightened affective response might also make those females more likely to be responsive to sexual cues in their offline environment, leading potentially to increased excitatory sexual approach behaviors. Such findings may also have implications for sexual risk behavior (Victor et al., 2015).

Females higher on erotophilia showed higher ratings of valence and moral and ethical acceptance, but less disgust for mainstream pornography. Similarly, more erotophilia was associated with more moral and ethical acceptance and less disgust for paraphilic pornography. Erotophilia was, however, not associated with how arousing the scenes of dominance, submission or sexual violence were perceived by the females. Other studies in Hispanic populations reported that erotophilia was positively associated with subjective sexual arousal (Arcos-Romero et al., 2019; Sierra et al., 2017). This study could not find such an association, which could be related to the way of assessing arousal, using SAM-rating scales (Bradley & Lang, 1994). These scales are non-verbal and do not specifically refer to sexual arousal, but arousal in general. Nevertheless, erotophilic sexual attitudes seem to be associated with the subjective experiences of valence, disgust and moral and ethical acceptance when females are exposed to pornographic stimuli. Further examination into how individual differences in erotophilic sexual attitudes affect approach or avoidance behavior toward sexual stimuli in females may also help illuminate perceptual processes that underlie sexually compulsive behavior or avoidance of sexual stimuli related to sexual dysfunction.

Additionally, related to hypersexuality, only the subscale assessing sexual self-control showed a significant association, and only with ratings of moral and ethical acceptance to mainstream pornography. The inability to control one's sexual behavior often leads to negative consequences for the individual (Klein et al., 2014; Yeagley et al., 2014). Interestingly, in this study, females that lack more self-control in sexuality-related behaviors perceived mainstream pornography as less ethically and morally accepting, but no such association was found with paraphilic pornography. In line with these findings, hypersexual men seem to hold negative attitudes toward pornography use and evaluate their own sexual morality more negatively (Štulhofer et al. 2016). These findings suggest that hypersexual tendencies and, specifically in females, a lack of self-control in sexuality-related behaviors, may have negative effects on one's moral and ethical acceptance in sexual contexts. Higher pathologizing interpretation of personal sexuality may relate to internalized moral and ethical stigma related to sexual conduct.

Further, females with higher ratings regarding problematic pornography consumption also rated mainstream and paraphilic pornography as more pleasant. The association between the obtained PPCS-6 ratings and higher valence toward pornographic material could be partially explained by the activation of the dopaminergic reward system, commonly used to explain clinically relevant pornography use (Stark & Klucken, 2017). According to this theory, pornography, as a natural reward, should activate the dopaminergic reward system already in sporadic users. Cue reactivity as a result of previous conditioning processes is believed to be greater in those with a more problematic consumption pattern, which may explain the positive associations with ratings of affective valence that were found. Accordingly, more frequent pornography users rate sexual material as more pleasant than less frequent users (Kunaharan et al., 2017), which was also observed in this study comparing females who have never consumed pornography with those who have. It has also been noted that when viewing pornography frequently, some individuals switch to viewing more intense material in order to maintain the same level of arousal, and habituation effects are thought to occur (Kühn & Gallinat, 2014), which may explain why higher scores on problematic pornography consumption tendencies are associated with more valence for mainstream as well as highly significantly associated with paraphilic pornography. Nevertheless, within this sample less than 2% of respondents had reported actual problematic pornography use according to PPCS-6 cutoffs (Bőthe et al., 2021), which is close to the results found in a German sample, where approximately 3% of the females exhibited problematic pornography use (Baranowski et al., 2019). Even though the majority of the sample did not show actual problematic pornography use, tendencies toward salience, tolerance, mood, modification, relapse, withdrawal, and conflict with pornography use were measured (Bőthe et al., 2021) and may give important insights into female pornography experiences in this regard and its associations with the affective evaluation of mainstream and paraphilic pornography.

No significant associations between sexual disgust sensitivity and arousal ratings were found. These findings regarding sexual disgust sensitivity are not consistent with those of Koukounas and McCabe (1997), who found a negative correlation between subjective sexual arousal in response to erotic videos and self-reported disgust sensitivity. As previously mentioned, this may be related to the use of different assessment scales for affective arousal. Sexual disgust sensitivity was, however, positively associated with ratings of valence and disgust for mainstream pornography. Surprisingly, significant associations with sexual disgust sensitivity were only found for valence and not for disgust ratings toward paraphilic pornography, even though the female respondents reported being more disgusted by, and less accepting of, paraphilic pornographic content. Additionally, sexual disgust sensitivity does not seem to affect moral and ethical acceptance of pornography, as no significant associations between sexual disgust sensitivity and this affective rating scale neither for mainstream nor for paraphilic pornography were found. Because disgust sensitivity has multiple motivational, maladaptive and adaptive functions (Curtis et al., 2011; de Jong et al., 2009, 2010; Oaten et al., 2009), assessments of disgust sensitivity might reflect drivers of decision making. Thus, females with high disgust sensitivity find mainstream and paraphilic pornographic images more unpleasant, which in turn may affect their likelihood of seeking such behaviors in offline settings. Understanding sexual disgust sensitivity as a driver of sexual decision processes may have clinical relevance, and it should be further explored to what extent and under what circumstances sexual disgust sensitivity acts as an inhibitor or reinforcer of sexual behavior.

### Psychological Intrapersonal Variables

In this female sample, no associations with negative affect or depression were found, neither for mainstream nor for paraphilic pornography. Of all the psychological intrapersonal variables examined, only anxiety showed a significant association, and that was with paraphilic pornography. The found association indicates that more anxious females perceive pornography of submission, dominance and sexual violence as more disgusting. In experimental settings, it has been demonstrated that induced anxiety can produce increases in reported disgust (Marzillier & Davey, 2005). Also, individuals that are high in anxiety symptoms report significantly more disgust than those low in anxiety symptoms (Olatunji et al., 2007, 2017). Since paraphilic pornography scenarios feature riskier and thus more costly sexual behaviors, this may explain why this relationship between fear and disgust was found only for paraphilic pornography. Given that pornography use is often postulated as a means of emotion regulation, a link to increased negative emotions, depression and anxiety with the affective perception of pornographic content would not have been precluded (Castro-Calvo et al., 2018; Paul, 2009; Willoughby et al., 2019). Nevertheless, in the context of paraphilic pornography, it is important to mention that, for example, individuals with sexual interest in BDSM, sexual practices that involve dominance, submission, and control, usually engage in them consensually and are, furthermore, similar to community samples in terms of depression and anxiety (Connolly, 2006; Dunkley & Brotto, 2018; Moser, 2020; Wismeijer & van Assen, 2013).

### Implications

Previous studies to the affective perception of sexual stimuli have been conducted with mainstream pornography and primarily with male participants. These studies provided insights into the basis of human sexual response, focusing on the underlying emotional mechanisms. However, current evidence shows a growing interest in sexual content, including depictions of violent sexuality, especially the more subtle sexual violence, in community samples. Acceptance and endorsement of sexual violence need not come only from the male side; acceptance from the female side can also play an important role in this matter. Given the sparse evidence on how females would perceive such content, and which intrapersonal variables might associate with such perceptual processes, this study offers further insight into emotional responses to diverse pornography scenarios from a female perspective. Such knowledge is helpful as emotional responses to a stimulus are said to highlight core mechanisms underpinning individuals’ behavior such as decision making regarding that stimulus situation (Emmons et al., 1985, 1986; Lerner et al., 2015). As emotions drive us toward goal-oriented actions, preventing harmful or negative social interactions, or promoting pleasant and positive social interactions (Lang et al., 1998; Mirabella, 2018), research on emotional responses to non-violent and violent forms of pornography may contribute to our understanding of individual and social responses to and mechanisms underlying the acceptance and seeking of such scenarios. Intrapersonal differences that lead females to perceive different types of sexual media as more arousing, pleasurable, disgusting or acceptable could also provide a predictor of the enjoyment and acceptability of such scenarios in an offline setting. Especially in the context of violent pornography, concerns have been raised, that the more frequently individuals are exposed to such depictions of sexuality, and the more accepting they are of such, the likely it might become that these types of scripts are also influencing dating and sexual relationships. Thus, understanding intrapersonal differences could be important from a female perspective of risk prevention. Additionally, understanding females perception of sexual media might also support greater acceptance of female sexual pleasure, which is particularly important in Hispanic countries where sexual double standards are still very present and traditional values may lead to the underestimation of the female sexuality (Giménez-Garcia et al., 2020, 2021). Additionally, knowledge regarding individual differences related to sexual perceptual processes can give clinical insights into the understanding of sexual dysfunctions.

### Limitations and Future Directions

This study shows limitations in terms of its sampling population. Firstly, older females also participated, which may have effects on the homogeneity of the observed results (Linden & Hönnekop, 2021).

Another limitation relates to the types of stimuli presented. In this study, the paraphilic images mainly showed humiliation or suffering of one partner, whereas the mainstream pornography showed a variety of sexual practices (masturbation, oral sex, vaginal sex, anal sex, group sex). It also makes a difference whether a person has a paraphilic interest in simulated or acted out rather than actual sadistic or masochistic activities (Moser, 2001), yet this study failed to ask participants about such differences in their preferences. In addition, calling certain sexual preferences paraphilic has been criticized (Herek, 2004; Inglehart et al., 2017; Joyal et al., 2015; Moser & Kleinplatz, 2020; Stefanska et al., 2022). Furthermore, the images contained scenarios with same- and opposite-sex porn actors and actresses, which may have influenced the results. Indeed, the correspondence between identity and sexual attraction is known to vary more among females than males (Chivers et al., 2010). We found such variability in our sample as well, with about 60% of females reporting that they were also attracted to same-sex, not just opposite-sex, individuals, even though nearly 70% reported being heterosexual. In general, females appear to have lower so-called category specificity with respect to sexual material, which is also observed in terms of their physiological responses (Chivers et al., 2010; Ziogas et al., 2023). Limitations, nevertheless, arise from the combined image categories under the term of mainstream pornography as well as an unbalanced female sample for different sexual orientations and relationship statuses. Research into relationship statuses should further investigate attachment-related affective dynamics (Sadikaj et al., 2011) in relation to the affective perception of mainstream and paraphilic pornography. It is not only intrapersonal dispositions and affective states, but rather a complex interplay of those with internalized social messages, values, interpersonal variables and experiences that contribute to how pornography is experienced by females (Ashton et al., 2018). Henceforth, investigations should explore more in-depth the diverse female experiences with pornography.

In addition, the cultural restriction to Hispanic females with a high level of education has to be noted. Findings may be influenced by sexual double standards and cultural stigma associated with the different content, as sexual practices that do not conform to the norm are generally more stigmatized (Digoix et al., 2016; Giménez-Garcia et al., 2020, 2021; Schmitt & Fuller, 2015). Consumption of pornography can lead to discomfort when there is a dissonance between the content of sexual material and one's sexual self-concept and values (Grubbs et al., 2019; Walters & Spengler, 2016). Thus, social context and social desirability may affect study outcomes in this domain, potentially preventing participants from providing honest responses. However, with regard to the here-obtained results, it was assumed of a more accepting female sample that is quite morally and ethically open to sexual material, received a rather open sexual education, and may have responded accordingly. The educational background of the sample could account for their high moral and ethical acceptance of any type of pornography (Kozloski, 2010).

In this study, care was taken to provide participants with an environment in which they were exposed to pornographic stimuli that is very similar to the typical setting of private pornography consumption, from the personal sphere of their homes and private electronic devices. Future studies should consider investigating female pornography consumption using momentary assessment, to provide them with a more natural feeling and be less likely to affect their ability to become aroused by it. The questionnaires in this study were, however, presented after the affective ratings were completed, so that participants had seen all the images before completing the questionnaire assessment. This may have led to a bias in the questionnaire data. Moreover, only subjective questionnaire data, which are susceptible to biases (Koukounas & McCabe, 2001; Seto, 2007), are available.

Emotional and sexual responses are always accompanied by activation patterns of body and brain (Janssen et al., 2007; Ziogas et al., 2023). Thus, data on psychophysiological responses to the pornographic stimuli would further enrich the study. Such additions would provide important insights into the relationship between intrapersonal variables and affective perception of sexual material and their physiological correlates, which could lead to more applicable conclusions for understanding sexual dysfunction related to compulsive sexual behavior, arousal difficulties, lack of sexual desire, and other persistent problems with sexual functioning in females.

### Conclusions

The different affective ratings for mainstream and paraphilic pornography indicate different affective perceptions of these two types of sexual media by females. Moreover, the results suggest that females' affective perceptions of pornographic content are mainly associated with sexual intrapersonal variables than with the psychological intrapersonal variables of affect, depression and anxiety. Sexual sensation seeking, erotophilia, sexual control, pornography use and sexual disgust sensitivity appear to be important for females in their evaluation of pornographic content in terms of valence, arousal, disgust as well as moral and ethical acceptance. Female anxiety was only associated with the disgust perception of paraphilic pornography. There is a need to better explore and understand females' experiences with pornography, particularly in Hispanic countries such as Spain, where there is little research in this regard and a sexual double standard still influences sexual behavior (Giménez-Garcia et al., 2020, 2021). Future research should include further variables of intra- and interpersonal value in order to offer a bigger picture of females perception of pornography and its relationship to sexual and psychological variables.

## Data Availability

All reasonable requests for data and materials will be met.

## Code Availability

All reasonable requests for code relevant to the analyses of this paper will be met.
